# Maintaining cognitive gains in type 2 diabetes: Barriers, supports, and patterns of strategy use

**DOI:** 10.1371/journal.pone.0349848

**Published:** 2026-10-05

**Authors:** Heather E. Cuevas, Jeeyeon Kim, Shenell T. Wood

**Affiliations:** 1 The School of Nursing, The University of Texas at Austin, Austin, Texas, United States of America; 2 Department of Physical Medicine and Rehabilitation, The University of Michigan, Ann Arbor, Michigan, United States of America; 3 Division of Allied Health, Victoria College, Victoria, Texas, United States of America; King Saud University / Zagazig University, EGYPT

## Abstract

In this study, we report how adults with type 2 diabetes maintained cognitive strategies learned in the Memory, Attention, and Problem-Solving Skills for Diabetes (MAPSS-DM) intervention. We identify three patterns of strategy maintenance that emerged from follow-up interviews with 19 participants: sustained use, partial use, and discontinuation. Factors that supported long-term use of strategies include consistent routines, social support, and personal value placed on cognitive improvements. Barriers include inconsistent routines, lack of support, and negative emotions such as guilt. This study emphasizes the need for personalized, adaptable cognitive interventions and ongoing support to promote lasting benefits in both cognitive function and diabetes self-care.

## Background

In the United States, over 40 million adults have diabetes (DM). Among those diagnosed with DM in midlife, decline in cognitive function over time is greater than in adults without DM [1,2]. Indeed, DM increases the risk for Alzheimer’s and related diseases (ADRD), and in those without a diagnosis of DM, changes in brain structure and activation patterns associated with insulin resistance and with obesity demonstrate that progression to type 2 diabetes (T2DM) may come after changes in cognitive function have started [3]. Detrimental structural and functional brain alterations have been found in those with T2DM [4], and T2DM may accelerate the rate of processes that increase the risk for cognitive impairment.

A number of modifiable risk factors for complications of T2DM (e.g., lifestyle, and social habits) are modifiable risk factors for cognitive impairment as well [5,6]. Many complications of T2DM can be prevented through modification of risk factors, and longitudinal observational studies suggest that the risk of T2DM complications is reduced in individuals who are physically active [7], are socially engaged [8], and eat a heart-healthy diet [9]. These behaviors are mirrored in guidelines to maintain brain health and prevent cognitive impairment [10].

Evidence shows that cognitive rehabilitation training can increase use of new cognitive skills and cognitively stimulating activities, resulting in improved neuropsychological functioning among those with chronic illnesses. Specific cognitive strategies or activities include activities to enhance problem solving (e.g., breaking tasks into smaller activities) or memory (e.g., mnemonics and mental snapshots) and strategies to manage stress (e.g., meditation). For example, the Advanced Cognitive Training for Independent and Vital Elderly (ACTIVE) study showed that cognitive rehabilitation training improved engagement in activities as well as memory in an elderly sample (*N* = 2,802) [11]. The Improvement in Memory with Plasticity-based Adaptive Cognitive Training (IMPACT) trial, one of the largest trials to test a cognitive training program to determine whether cognitive abilities could be improved, showed positive changes in day-to-day life [12].

Current surveys do not capture the full experience of continued use of cognitive strategies, whether because of participating in cognitive rehabilitation interventions or as a part of daily life. Strategies for cognitive domains other than memory are not assessed, and items included on standard surveys used to evaluate use of cognitive strategies or cognitive training may have different meanings or intensities across individuals and ethnic groups, indicating a need for further assessment [13,14]. For example, Wilson et al. [14] found a weaker link between reading and cognitive function in African Americans than in non-Latinx Whites. Further, prior work has shown that the use of cognitive strategies is often driven by individuals’ perceived cognitive difficulties rather than by objective test performance [15], suggesting that subjective experiences should be considered when assessing strategy use and designing interventions. Cognitively stimulating activities and strategies (e.g., smartphone applications, exercise programs) can also require financial resources, and they are affected by changes in life situations.

Our recent meta-synthesis [16] of the literature on experiences of cognitive dysfunction in the context of a variety of chronic illnesses (e.g., diabetes, chronic obstructive pulmonary disease) has revealed one overarching theme, the process of adapting, as well as four sub-themes: (1) perceptions of self; (2) symptoms; (3) relationships; and (4) health care. Participants in the reviewed studies used a wide number of cognitive strategies; but more evidence is needed to inform effective interventions, including qualitative information on how participation in cognitive rehabilitation interventions affects the use of cognitive strategies in day-to-day life.

Management of T2DM has long shifted from a model of passive clinical compliance to one of intensive, lifelong self-management. While clinical guidelines clearly define therapeutic targets, a primary challenge in T2DM care is sometimes less identifying what patients need to do but ensure they continue doing it over time. Clinical interventions frequently succeed in initiating short-term lifestyle changes, yet a steep decline in adherence typically occurs several months [17]. This widespread failure to sustain lifestyle modifications highlights a critical gap in current behavioral interventions: the transition from conscious, effortful execution to permanent behavioral integration. Strategy maintenance involves equipping individuals with the cognitive flexibility to navigate real-world disruptions, preventing minor relapses from turning into total behavioral abandonment.

### Purpose

In this study, we explore the how participants with T2DM in a cognitive rehabilitation intervention used cognitively stimulating strategies to support DM self-management (DMSM) at 3-month follow-up. We examine their subjective experiences and patterns of cognitive strategy maintenance that emerged from interviews, which include facilitators and challenges related to continued use of the cognitive strategies.

### Research questions

1. How do participants in a cognitive rehabilitation intervention integrate cognitive strategies in their day-to-day lives?
2. What are the barriers and facilitators related to maintaining the use of cognitive strategies for people with T2DM?

### Theoretical context

The Scaffolding Theory of Aging and Cognition–Revised (STAC-r) provided our theoretical framework for examining the experiences of individuals participating in the intervention [18]. The STAC-r posits that age-related changes in the brain, such as loss of brain volume, are a main cause of cognitive decline. Compensation for this decline is bolstered by scaffolding through the use of alternative brain regions and/or the generation of new neurons. This scaffolding is reinforced by positive daily routines and experiences as well as participation in cognitively stimulating activities. Interventions such as cognitive rehabilitation and training as well as social and physical activities can improve scaffolding and increase compensatory abilities. According to the STAC-r, brain structure and function can be improved through interventions that increase brain stimulation and functional connectivity, especially in those with lower cognitive performance at baseline [19]. Even with injury or decline, it is possible to maintain, if not improve, cognitive function [18]. In the Dallas Lifespan Brain Study [20], even some older adults with decreased memory retrieval relative to younger adults had more “youthful” activation patterns than did others of the same age. This was thought to be consistent with compensatory scaffolding and functional brain maintenance, because there was more activation outside core task regions.

## Methods

### Design and study sample

The data in these analyses are from a larger longitudinal study to evaluate the effectiveness of a comprehensive cognitive rehabilitation intervention for people with T2DM: Memory, Attention, and Problem-Solving Skills for Diabetes (MAPSS-DM). The present qualitative study is based on the 3-month follow-up data from the MAPSS-DM trial. For this study, we have adopted a qualitative descriptive approach in order to learn from participants and understand their unique perspectives regarding the intervention. The data consist of individual interviews with participants (*N* = 19) enrolled in the intervention group. Recruitment for the qualitative portion of the study started in September 2023 and ended in September 2024. Invitations were sent to all participants who completed the intervention in the main study after the 3-month follow-up visit, explaining the interview purpose and time commitment. Control group participants were not invited as the purpose of this portion of the study was to evaluate maintenance of strategies in the intervention. Written informed consent was obtained at the start of the study. Initially, 23 participants indicated they would be willing to be interviewed; however, only 19 responded for scheduling. All participants provided written informed consent prior to enrollment in the study. The study was approved by the University of Texas at Austin’s Institutional Review Board (IRB #00000464).

### MAPSS-DM intervention

The MAPSS-DM intervention is an 8-week cognitive rehabilitation program with webinar group sessions to increase knowledge and build self-efficacy for new cognitive compensatory strategies via individual online home-based practice of those skills. A pilot randomized controlled trial with adults with T2DM (*N* = 70; mean age 65.2 ± 3.4 years; 51% non-Hispanic white; mean A1C 8.3%) evaluated the efficacy of the MAPSS-DM intervention to improve overall cognitive function and DMSM [21]. Four online sessions were held every other week for 8 weeks. Classes 1 and 2 focused on common cognitive problems in T2DM and strategies to improve cognitive skills. Classes 3 and 4 focused on lifestyle changes to support cognitive functioning and self-management. The home computer-training portion of the intervention, “brain games” developed by Posit Science, were adapted to users’ performance via integrated hierarchical structures [22]. Participants were asked to practice the online games for 20 minutes, 5 days a week.

### Interview design

The interview guide for the intervention was based on reviews of the literature on cognitive rehabilitation and DMSM interventions as well as our prior research examining participation in the MAPSS-DM intervention [23,24]. This semi-structured interview guide (Table 1) enabled us to explore participants’ experiences in using cognitive strategies and maintaining DMSM activities. Questions covered topics such as (1) a typical use of DMSM; (2) use of cognitive strategies and how they did or did not help with DMSM; and (3) reflections on continuing those cognitive strategies over the long term. Because the interview guide was kept broad it allowed for participant-driven exploration and encouraged in-depth authentic narratives. Nineteen interviews were conducted by the study’s primary investigator at the 3-month follow-up visits in the MAPSS-DM study. To reduce influential bias, status, function and hierarchical position of the PI were not introduced to participants and visual indication of status was purposely removed (e.g., lab coat). No participants were under the care of the PI, and it was explicitly mentioned that participation in the study was disconnected with care. The interviews ranged from 45 to 75 minutes in length and were audio recorded in a private room at a university clinic or over Zoom. Field notes were taken by the interviewer. Interviews were transcribed verbatim and anonymized.

**Table 1:** Interview Guide.

Theme	Questions
**A typical day during the intervention**	Can you try to describe what a typical day was like for you in the last three months? • What time did you get up? • When did you eat meals? Did you eat with others? • How about work? • Have you been doing any leisure time activities? • Did you miss any medications? • Have you been doing your regular activities at home? • Do you think you were more/less forgetful (or less attentive, or had slower thinking)?
**Experiences with performing cognitive strategies**	Now that we’ve reviewed a typical day for you, can you tell me more about how it has been to use the strategies you learned in the classes? • What has been easy? • What has been challenging? How have the cognitive strategies fit in your daily routines in the last 3 months? • Have you changed anything in your daily life or diabetes management to use them? • Have you missed out on anything because of them? • Have others reacted to your use of them? How? Did you ever consider not using them? • When? • What kept you going? What do you think about daily life or diabetes management now compared to before the intervention?
**Thoughts on maintaining cognitive strategies**	Have you thought of ways to keep using the cognitive strategies now that the intervention is over? What are your overall thoughts on cognitive strategies now that you have used some?

### Data analysis

Qualitative data analysis enabled us to consider participants’ experiences of their maintenance of cognitive strategies, as well as facilitators and barriers related to its success. Specifically, Braun and Clarke’s six-phase thematic analysis framework and inductive reasoning was used [25]. This is a flexible method designed to operate in an essentialist (realist) epistemology in which the focus lies on participants’ lived experiences and assumes that language can reflect meaning in a relatively straightforward way [25]. First, after interview transcripts were uploaded to NVIVO software for qualitative data analysis, they were read independently by two researchers in order to obtain insight into use of cognitive strategies and DMSM (step 1: familiarization with data); meaning units (e.g., sentences, paragraphs) describing participants’ experiences with the intervention were identified, summarized, and collapsed into codes (step 2: generating initial codes). Facilitators and challenges related to both DMSM and cognitive strategy maintenance were also identified. After codes were sorted, themes were identified with the empirical codes (step 3: searching for themes). Themes were reviewed for coherence and distinction, with some codes merged or discarded (step 4: reviewing themes). The remaining themes were then refined and described in relation to the research questions (step 5: defining and naming themes). Finally, the themes were interpreted and linked to the literature (step 6: writing the report). Repeated review of the findings and transcripts verified categories that emerged from the interviews (Table 2). Throughout the process, both semantic and latent approaches were applied with codes and themes identified at the explicit, surface level as well as looking for underlying subtext, ideas, and assumptions that informed the sematic content.

**Table 2:** Examples of Analytic Categories and Descriptive Themes.

Broad groups of analytic categories	Analytic categories	Examples of descriptive themes based on code groups	Exemplar quotes
**Maintenance patterns**	• Practice of at least one cognitive strategy • Practice of 2–3 cognitive strategies • No longer used cognitive strategies	• Number of cognitive strategies per cognitive domain • Help with practicing strategies • Integration of strategies • Enjoyment of strategies	“I only used one strategy a day. I wanted to focus on my attention span.” “I found an app to use the planning strategies more.” “I liked the strategies to avoid multitasking. The others seemed obvious.”
**Routines predicted strategy use**	• Consistent daily routines • Habit formation • Varied daily routines	• Routines made strategies easy • Temporary vs. permanent strategy use • Work status • Holidays, weekends disrupted strategy use	“Practicing the strategies was easy Monday – Friday. On the weekends, it was harder because of family.” “Once I got the hang of [the executive function strategies], it was easy to keep up.”
**More than a glucose goal**	• Glucose as a goal • Rewards of strategy use • Glucose as the only goal	• Outcomes other than glucose • Internal rewards for cognitive strategy use • Focus on glucose as the primary outcome • Anticipated outcomes	“I thought [the cognitive strategies] would fix my glucose. But they did more than that.” “It felt good to focus on my brain health. I think that will only help my sugar in the long run.” “My glucose is great. This helps a lot with self-management and my sugar is better.”
**Flexibility**	• Positive attitude • Strategies seen as intrusive • Removing feelings of guilt	• Intrusiveness of the intervention • Prioritization of activities to include strategies • Social contexts of strategy use • Feelings of guilt for not using strategies • Perceived disappointment of research staff • Long-term mindset	“It took a lot of time to play the games. But I wasn’t 100% sure I’d be allowed to stay in the study if I didn’t do them.” “At first I felt bad I couldn’t keep up [with the games], but it ended up easy to do when I was waiting in line or at the doctor’s office.” “Guilty or not for not doing the strategies. It ended up fun.”
**Social support**	• Adapting to other’s needs • Disruption of family routines • Social network support	• Support from family • Support from friends • Support from health care providers • Technology	“My wife kept bothering me to do [the strategies] -in a good way. So, I did.” “The games were easy to do on my phone once my grandson set it up.” “My doctor was very happy when I told him about the study. He said it was good practice.”

We employed multiple strategies in ensure credibility, transferability, dependability and confirmability. We also applied active reflexivity including recognizing the researchers’ positionality, the participants’ possible perceptions of that positionality and the assumptions made about those perceptions into the interviewing and coding [26]. To improve comprehensiveness, the consolidated criterion for reporting qualitative research (COREQ) checklist was used for reporting the data (see Supporting Information in S1 Fig) [27].

Sample size adequacy was evaluated using a dual framework combining a model of information power and the principle of data saturation [25,26]. Regarding information power, the study had relatively narrow and specific aims (exploring intervention integration and its barriers and facilitators) and a highly specific sample (19 participants who completed a distinct cognitive training intervention and who were rich information holders). The interview dialogue quality was strong, facilitated by the semi-structured interview guide and the cross-case analysis strategy focused on patterns within this cohort. Because of these characteristics, a moderate sample of 19 was considered sufficient. Additionally, data saturation was evaluated continuously. By the final three interviews no new codes, thematic properties, or novel barriers/facilitators emerged, confirming that informational redundancy had been reached.

## Results

### Participants

All participants had completed the entire MAPSS-DM intervention and attended all the online sessions. They played the brain games 5 times a week on average, for 30 minutes each session. Their average age was 66 years; 52% were female, 58% were non-Hispanic White; and their mean time since diabetes diagnosis was 9.9 years (Table 3). The participants in the qualitative portion of the study were slightly older than participants in the larger project (mean age 59.6 years, SD 5.99). All other demographic characteristics were not significantly different between the two groups.

**Table 3:** Participant characteristics (n = 19).

	n (%)	Mean (SD)	Range
**Gender**			
**Male**	9 (47.4)		
**Female**	10 (52.6)		
**Ethnicity**			
**Non-Hispanic White**	10 (52.7)		
**Hispanic**	6 (31.6)		
**Black**	3 (15.7)		
**Age**		66 (5.7)	56 - 74
**Years with Diabetes**		9.9 (5.5)	4.6–12.1
**A1C**		8.7 (3.2)	7.2–10.14

### Maintenance patterns

Three patterns of cognitive strategy maintenance emerged. First, 10 participants continued to practice at least one cognitive strategy per cognitive domain (e.g., memory, attention) after the intervention. This was defined as “sustained use.” Six participants continued to use 2 or 3 strategies total (partial use), and 3 participants were no longer using specific cognitive strategies (discontinuation of strategies) taught in the intervention. In all the interviews, participants stated that they wanted more attention and help in using the strategies in the beginning of the intervention. But by the end of the intervention, those still using at least 2 strategies said they had made them “routine.” One said, “I don’t even have to think about them anymore. I’ve made it part of how I do things. If I need to remember things, I make a list. I turn off notifications on my phone if I’m working. I actually like doing the 10-minute meditation in the morning.” However, events such as illness and family obligations were barriers to continued use of cognitive strategies. As one put it, “I think it was tough. I don’t know if I did a good enough job of trying to make it seamless and put these strategies into what I do every day. So, when [family member] got sick, I just dropped it all.”

### Routines predicted strategy use

Participants who already had consistent daily routines during the intervention thought that the additional cognitive strategies made “life overall as well as doing all the diabetes things” easier. Using the cognitive strategies seemed to depend on the participants’ ability to actively adapt them to their preexisting rhythms, so that the strategies became less a temporary regimen and more a practice of daily life. For example, one man said that “I think I’ve made it [a cognitive strategy] a part of what I do; it’s not that hard, because it’s actually helpful.” It is possible that it was easier for this person because he was retired; he was still very active, but he had relatively few non-negotiable demands on how he organized his life—as opposed to other participants who still had 9-to-5 jobs. However, others who were still working full time mentioned that the routine of their schedule allowed for consistency and for fitting strategies in until they became habits. As one woman put it, “Yeah, I’m busy, but work is work, it will always be there. I have to use the time I have and see how I can make good choices within that time.”

When daily routines after the intervention varied, however, cognitive strategy maintenance until the 3-month follow-up was perceived to be more difficult. For example, “There is no routine. I think that explains why I can’t keep them [cognitive strategies] up. I’ve never been good with taking care of diabetes consistently so I’m not sure how I can be good with adding something.” Similarly, some found it difficult to continue strategy use on the weekends or holidays. More irregular patterns in daily life were associated with less attention to diabetes management and less use of cognitive strategies.

### More than a glucose goal

For participants who used more cognitive strategies, having a set glucose goal (e.g., “lower my A1C to 7%”) was not stated to be the driving force for strategy use, because they prioritized other outcomes. For example, some said that it was important to avoid multitasking. This allowed them to focus on one task at a time and “enjoy it more.” One woman put it thus: “I’m cooking. Yes, this kind of has to do with my sugar because it’s food, but it also gets me out of my head and I can think more about what I’m doing, not make a mistake, and the food is better. I think that carries over to more than just one thing at a time.”

Using the strategies was also seen to be rewarding because, as one participant said, “I’m more in touch with what my body wants.” Examples included getting better sleep by not staying up past a certain time (“this helped me pay better attention during the day”) or feeling more energized: “My blood sugar, which has been crazy, has been much more stable. I can feel that now. So, I’m pretty sure I’m less stressed. And the CGM shows that, right?”

Participants who did not maintain cognitive strategies explained that they were very focused on achieving better A1C or better glucose readings at home. When they did not have what they thought to be improved glucose levels, they struggled to maintain the recommended strategies. Cognitive strategies’ longer-term use was still influenced somewhat by anticipated and experienced glucose outcomes.

### Flexibility and feelings of guilt

Most participants had a positive attitude toward using cognitive strategies but often considered the intervention’s “imposed” regimen to be somewhat intrusive in the short term until they found ways to “make it work” and fit select strategies into their daily lives. Examples included moving a morning meditation to after lunch, taking the weekend off from making to-do lists, and adding smartphone apps to help with alarms/reminders. One person elaborated on her regimen thus: “When I’m home there’s more activities I can consider, but when there are other times when I have to know what other people—my family, work—want, it was hard at first to make use of strategies. Some I tossed out and others I kept.” Those who successfully continued cognitive strategies used the elements that worked for them and made adjustments that they felt would help over the long term.

The example in the preceding paragraph illustrates the dependence of successful maintenance on social contexts. All participants said they had some initial difficulty in maintaining strategies because they feared “letting others know I might not be as sharp as I could be.” However, how participants coped and adapted were seen to be central components of success. When feelings of guilt or “shame” were avoided, strategies were maintained. But for two of the participants, not maintaining strategies in potentially embarrassing situations led to guilt. As one woman said, “I went to the doctor and forgot my list of medications and my sugar book [logbook of home glucose readings]. I knew I had ways to help remember these things, but I got lazy and didn’t use them. I got a guilty conscience because I didn’t need to forget them.”

Feelings of guilt also influenced participants’ motivation to continue using cognitive strategies. One participant felt guilty for letting down the project’s staff by not meeting prescribed amounts of computer training. This participant’s perceived lack of flexibility or unpreparedness led to self-disappointment. A few said that it was like “all the times I’ve failed to lose weight.” Some said it was as if they had failed at “just one more thing related to my health.”

### Social support as key to success

Most participants stated that a major driver of successful maintenance was family support and the ability to adapt strategies to families’ needs. Participants whose families helped with strategies reported more success (e.g., “remind me to set an alarm”). On the other hand, a lower level of support decreased use. Family members would interrupt or blame participants for taking too much time with strategies—especially strategies that seemed to disrupt established routines. For example, one participant wanted to video record a meeting so she could review it later, but a co-worker objected. Although she was initially discouraged and wanted to stop trying to find a solution to help with memory issues at work, she instead found a notetaker smartphone app that made it easier to keep a visual and audio track of meetings. Impatience from family members was also an impediment to maintaining strategies. One participant, who mentioned that it took her longer to complete tasks because she wanted “to be thorough and make sure I don’t forget anything,” said that this seemed to frustrate her husband, and so she would revert to hurrying through things even if it meant forgetting something.

Overall, however, participants said that their social networks were supportive even if sometimes their health care providers were not. Participants wanted more support from health care providers and follow-up to help continue good health practices, which included cognitive strategies. As one woman said, “You do it on your own, but it would have been nice to have more than just the study staff calling to check on you. It is easier if you need to be accountable to someone in your real life.”

## Discussion

This is one of few studies to examine the experiences of people with T2DM in maintaining cognitive strategies learned in a comprehensive cognitive rehabilitation intervention. Most participants in the intervention continued using some of the cognitive strategies that they learned and adjusted them to fit within their day-to-day lives. Their interviews indicated that several facilitators and barriers related to cognitive strategy maintenance were important to address. Facilitators included consistent day-to-day patterns, active adaptation, outcomes in addition to glucose levels, avoiding guilt, and social support. Challenges included social events, inconsistent daily rhythms, irregular schedules, preoccupation with glucose control, low levels of social support, and self-blame when not meeting goals.

Consistency was a main driver for maintaining strategies. If cognitive strategies were easily added to daily responsibilities, including those for work and family, participants said that they were easier to continue over the long term. In other cognitive rehabilitation intervention studies, this has also been the case [28,29]. Thus, there were commonalities with other lifestyle interventions, because participants faced similar challenges as they embedded strategies into the rhythm of their day-to-day routines.

Perhaps participants who struggled to maintain cognitive strategies had not received adequate guidance and support for integrating them and forming new habitual behaviors. One improvement of the intervention might therefore be to increase its personalized guidance and support. Future iterations of MAPSS-DM could focus more on adapting strategies to fit individuals’ daily challenges. According to STAC-R, sustained strategies or interventions can fundamentally strengthen cognitive reserve. Providing tailored support helps transition these strategies from effortful, temporary scaffolding into stable, automated habits that require fewer cognitive resources. Participants might benefit from more discussion with other participants, because their shared experiences could be helpful by increasing support and strategy use.

Flexibility for these participants resulted in greater maintenance of cognitive strategies. Most participants adjusted the strategies that they did use and how they incorporated them into their daily lives during the follow-up period and said that they might continue to adjust them as they moved forward. This type of flexibility or adaptation, which is needed for long-term success, has been described before in the behavior change literature. “Flexible adaptation” in the self-management of chronic conditions is associated with sustained use of healthy behaviors such as exercise and diet [16]. From a STAC-R perspective, this flexibility reflects the dynamic nature of scaffolding; compensatory strategies are not rigid fixes but adaptive behaviors that must evolve alongside changing environmental demands to effectively support cognitive health. On the other hand, rigidity or an “all or nothing” approach negatively influences self-management and healthy behaviors [30,31]. So far, the science of cognitive strategies does not emphasize “flexible adaptation” specifically as an integral part of long-term strategy use, but it has been conceptualized as consisting of easy-to-follow optional activities rather than as a single fixed approach [32]. The findings of our study show how important it is for people to actively make changes and incorporate or adapt strategies over time.

Many of those who maintained strategies were content with achieving a subjective outcome, such as being more organized, rather than focusing on them solely to lower glucose levels. Prior work has described satisfaction with results as essential for long-term success [33,34]. Although cognitive strategies have not been conceptualized as a means for glucose control in many other qualitative studies [24,25], our results also show that for some participants, an improved sense of well-being was a valuable outcome. It may be useful to investigate whether cognitive strategies should be conceptualized as activities to fulfill a variety of outcomes.

Whether or not the cognitive strategies disrupted social habits or had family support also affected maintenance. Other studies have found better adherence to positive health behaviors with higher levels of social support [35,36]. This aligns with the STAC-r, which posits that social engagement and cognitive strategies act as vital compensatory “scaffolds” to support neurocognitive function in older adults. Any self-management change, be it the addition of cognitive strategies to support DMSM or another health behavior, can be challenging because it may disrupt already established patterns in habits with friends and family. Some activities may often conflict with social conventions. Therefore, some participants chose to set aside any positive health behaviors learned in the intervention at certain times without feeling guilty. And when they did, they found it easier to pick up those health behaviors the next day. Other studies have reported that positive health behavior maintenance is inversely associated with perceived shame, guilt, and failure [33,37]. Accepting the “ups and downs” of health behaviors positively influences maintenance [38]. Our results indicate the importance of avoiding self-blame when one is faced with new or more challenging situations. From a STAC-r perspective, managing emotional distress and planning for these disruptions protects the individual’s motivational and cognitive reserve, making the behavioral scaffolds easier to build. Planning for such challenges may improve maintenance. Supportive strategies (peer or professional) are a likely helpful addition to interventions and regular post-intervention care.

Being held accountable to family, friends, or health care professionals was helpful for the continued use of cognitive strategies. Incorporating follow-up appointments with health care providers into cognitive rehabilitation interventions may be helpful. Because the goal of such interventions is to integrate different behaviors in daily life, rather than answer to health care providers or research teams, this could be a short-term intervention component, with continual guidance until new habits involving cognitive strategies are better established.

### Strengths and limitations

To the best of our knowledge, this is one of the first projects to provide an in-depth examination into the maintenance of cognitive strategies for people with T2DM. The details of the study’s results strengthen the understanding of facilitators and barriers related to maintaining cognitive strategies in a population that has a burden of disease management behaviors such as DMSM. However, the study has certain limitations. Almost half of the participants were retired, over age 65, with no young children at home. Nevertheless, some of the results may be pertinent for other age groups. In addition, participants volunteered for the study and were considered “engaged completers.” Because the 19 participants successfully finished the intervention, their perspectives are inherently shaped by a baseline level of motivation, adherence, and perceived value. Consequently, the data yielded predominantly positive themes while barriers experienced by non-completers remain uncaptured. This survivorship bias limits the transferability of the findings. In usual care settings, people may be less motivated. However, this challenge suggests that barriers to employing cognitive strategies may be more common, and so we need to address them early.

## Conclusion

Cognitive strategies can be incorporated into real-life DMSM. In fact, for many people, the strategies already are—they are just not labeled as such. However, flexibility is needed for maintenance. Successful cognitive strategy maintenance was influenced by consistency in daily life, social support, and avoiding guilt or shame related to perceived failures. Including these facilitators in future research may increase integration of cognitive strategies in day-to-day life. Personalized support for adjusting cognitive strategies is needed, and studies should explore the effectiveness of incorporating personalized support into DMSM education programs.

## Supporting information

S1 Fig COREQ Checklist. COREQ (Consolidated criteria for Reporting Qualitative research) Checklist.
(PDF)
