# Supplementary material for: Maintaining cognitive gains in type 2 diabetes: Barriers, supports, and patterns of strategy use
Source: PLoS One. 2026 Oct 5;21(10):e0349848. doi: 10.1371/journal.pone.0349848 (PMC13637923; doi:10.1371/journal.pone.0349848)
Supplement: Supplementary file 2 [file pone.0349848.s002.docx]

| **Reviewer Comments** | **Responses** |
| --- | --- |
| **Editor** |  |
| Please include your full ethics statement in the ‘Methods’ section of your manuscript file. In your statement, please include the full name of the IRB or ethics committee who approved or waived your study, as well as whether you obtained informed written or verbal consent. If consent was waived for your study, please include this information in your statement as well. | Information was added (p. 6, lines 125-129; 131- 132. |
| **Reviewer 1** |  |
| The authors should provide more details about recruitment and sampling. It would be helpful to know if the four participants who agreed to be interviewed but ultimately did not participate differed from the nineteen participants included. Since all interviewed participants completed the intervention, the sample might represent a more motivated or adherent group. | We added detail to the study sample section (p. 6, lines 125-129). Additionally, we discussed the possibility of increased motivation for the current sample in the limitations section (p. 19, lines 384-385). |
| The analysis process should also be clarified. The manuscript mentions 88% agreement among the researchers, but it is unclear how this percentage was calculated or how disagreements were resolved. Additional details on coding would enhance methodological transparency. | Clarification was added to the Data Analysis section (p. 8-9, lines 167-190). Table 2 provides detail on how codes moved from participants’ words to themes to analytic categories, to broader themes. |
| The results section would also benefit from a clearer distinction between the three maintenance profiles: sustained use, partial use, and discontinuation of strategies. | Definitions of sustained use, partial use and discontinuation of strategies were added to the results section (p. 12, lines 208-212). |
| Some conclusions should be formulated with greater caution. The manuscript occasionally suggests that cognitive strategies improved diabetes self-management or glycemic stability, but the data presented are primarily qualitative and self-reported. These points should therefore be presented as participants' perceptions unless objective data are added. | The manuscript was edited throughout and detail specific to this concern was added to the Discussion section (p. 18, lines 345-352). |
| The STAC-r theoretical framework is relevant but could be better integrated into the discussion. It is well presented in the introduction but is underutilized in interpreting the results. | Detail regarding the STAC-r framework was added to the discussion section (p. 17, lines 322-328; p. 17, lines 336-338; p. 18, lines 355-357, 366-368). |
| Finally, the data availability statement should include a precise link to the repository, a DOI, or an access number. | Data is published at “MAPSS Qual Dataset.” Texas Data Repository. https://doi.org/10.18738/T8/DPNOYJ. |
| The authors should also check Table 3, particularly the ethnic categories and A1C values, and correct minor language and formatting errors. | Formatting was corrected and errors fixed. |
| **Reviewer 2** |  |
| Background: This part of the manuscript is heavily weighted toward broad biomedical background on diabetes-related cognitive decline, Alzheimer’s disease risk, and neurobiological mechanisms. While these areas are well cited, the framing becomes diffuse and at times reads more like a general narrative review than a focused rationale for a qualitative study. For example, extended discussion of gray matter changes, insulin resistance-related brain alterations, and multiple cognitive intervention trials across different populations dilutes the central aim of the study, which is not to establish the efficacy of cognitive interventions but to understand post-intervention maintenance behavior. As a result, the actual research gap, how and why individuals with T2DM sustain or discontinue cognitive strategies in daily life, emerges relatively late and remains under-specified. I suggest a more focused introduction that directly builds toward strategy maintenance, habit formation, and behavioral integration in diabetes self-management would strengthen conceptual clarity and better justify the qualitative approach. | The Background section was edited and information regarding habit formation and behavioral integration was added (p. 4, lines 77-86). |
| Material and Methods: The authors state they used a “qualitative descriptive approach” and Braun & Clarke thematic analysis, but do not explain whether the analysis was inductive or deductive, semantic or latent, or what epistemological perspective guided interpretation. The analytic approach currently feels vague and procedural. Example: “Themes were identified with the empirical codes” is too general. Please explain how themes were developed and refined. | Clarification and details were added to the Data Analysis section (p. 8, lines 167-190). Table 2 provides detail on how codes moved from participants’ words to themes to analytic categories, to broader themes. |
| The use of “88% agreement” is problematic in the context of Braun & Clarke thematic analysis. Reflexive thematic analysis does not typically rely on intercoder reliability statistics. This creates methodological inconsistency and suggests confusion between coding reliability approaches and reflexive thematic analysis. Example: “The researchers had a high level (88%) of agreement…” should either be justified more carefully or removed. | Reference to 88% agreement was removed. |
| Sampling and recruitment require stronger justification. Participants were recruited from intervention completers who agreed to follow-up interviews, which likely introduced selection bias toward highly engaged participants with more positive experiences. Please discuss: why 19 participants were considered adequate? whether thematic saturation or information power was considered? How non-participation may have affected findings? | We stopped the individual interviews and focus groups after 19 participants,  as we considered that saturation was achieved. We had specified in the protocol that data collection would continue until saturation point had been reached and no new domains or themes were generated (p. 12, lines 202-204). How non-participation affected findings was added to the limitations section (p. 19, lines 384-385). |
| The interviews were conducted by the primary investigator, but there is little discussion of how this may have influenced participant responses or interpretation of findings. Participants may have felt pressure to provide favorable responses because the interviewer was connected to the intervention study. | Detail was added: To reduce influential bias, status, function and hierarchical position were not  introduced to participants and visual indication of status was purposely removed (for example  lab coat and signs of academic position). No participants were under the care of the PI, who is an APN, and it was explicitly mentioned that participation in the study was disconnected with any type of medical care. (p. 7, lines 155-158). |
| The interview guide appears broad and occasionally unfocused relative to the stated aims. Several questions (e.g., wake-up time, meals, leisure activities) are only indirectly related to maintenance of cognitive strategies and diabetes self-management. | The interview guide was kept broad to allow for **flexibility** and **participant-driven exploration**. Rather than acting as a strict, rigid script, it served as a **memory aid** and thematic checklist. This openness prevented missing unanticipated, emergent themes and encouraged in-depth, authentic narratives. We have added more description of the interview guide to the manuscript (p. 7, lines 152-153). |
| More detail is needed regarding the actual analytic process used in this study: how codes evolved? how disagreements were resolved? how themes were refined? | A step-by-step guide to the analytic process has been added in the analysis section (p. 8-9, lines 167-190). Table 2 shows how the researchers moved from quotes to descriptive themes based on code groups to analytic categories to finally broad groups of analytic categories. |
| The themes appear largely positive and intervention supportive. It is unclear whether the analysis actively explored negative experiences, disengagement, or resistance to the intervention. | We have included quotes throughout the results section (pp. 12, 14, 15) from participants who did not continue to use certain behavioral strategies. We have also added some discussion as to how this means the intervention was “less successful” or needs to be adapted/refined (p. 16). |
| Participant characteristics for the qualitative sample are missing. Readers need demographic and clinical information to assess transferability of findings. You cite previous work ref number 25 and you blind it for the review process, regardless the demographic information is important and need to be stated in this paper. | Participant characteristics are outlined in Table 3. |
| The manuscript claims use of the COREQ checklist to improve rigor; however, COREQ is a reporting guideline rather than a methodological strategy. | We employed multiple strategies to ensure the credibility, transferability, dependability, and confirmability of this research, and applied active reflexivity, understood as aligning three levels of reflection: researchers’ positionality, participants’ perceptions of that positionality, and the assumptions we make about those perceptions and other factors built into the qualitative interviewing and coding process. Reflexive awareness of the team’s positionality, together with investigator triangulation during coding, was used to support transparency and rigor (p. 9, lines 186-190). |
| The methods section currently mixes quantitative concepts of rigor with qualitative thematic analysis in a way that weakens methodological clarity. Greater transparency and consistency are needed. | We have added detail to the methods section (also see the above response). |
| Results: |  |
| Authors need to be cautious in interpretation of what qualitative data can support. For instance, statements such as “consistency was a main driver for maintaining strategies” or “flexibility resulted in greater maintenance” imply causal relationships that cannot be established from retrospective interviews. The findings would be more appropriately framed as perceived associations or reported influences. Similarly, while participant counts (e.g., 10 sustained users, 6 partial users, 3 discontinued) are helpful for contextualizing variation, they are occasionally treated as meaningful distributions rather than illustrative patterns, which risks overstating inferential weight in a small purposive sample. | We have tempered the causal language throughout the results section where appropriate (p. 13, lines 223-224, 236; p. 14, lines 244, 250, 255; p. 15, lines 274. 282, 286). |
| Discussion: The discussion would benefit from tighter linkage between specific empirical themes and their theoretical implications, as well as clearer acknowledgment of the study’s limited transferability due to sample characteristics (highly motivated completers, older adults, and intervention participants only). | We have revised the discussion section and improved these linkages as well as expanded the limitations section (p. 17, lines 322-328; p. 17, lines 336-338; p. 18, lines 355-357, 366-368). |
| Limitations- Please address all issues that cannot be resolved in the method section here. Please discuss here – the dual role of PI as an interviewer too, and participants as both intervention completers and interviewees. And please address the sampling limitations. | We have expanded the limitations section as well as included more detail in the methods section discussing these issues (p. 6, lines 125-129; p. 7, lines 155-158; p. 19, lines 382-387). |
| Minor Concerns-Line 37: “find T2DM [4], and T2DM” fix the word and. | Corrected. |
| The paper currently too long, please try to shorten it and make it easier to read. | We have edited and shortened the paper where appropriate. |
