# Supplementary material for: Maintaining cognitive gains in type 2 diabetes: Barriers, supports, and patterns of strategy use
Source: PLoS One. 2026 Oct 5;21(10):e0349848. doi: 10.1371/journal.pone.0349848 (PMC13637923; doi:10.1371/journal.pone.0349848)
Supplement: Supplementary file 3 [file pone.0349848.s003.docx]

| Reviewer Comments | Response |
| --- | --- |
| Add an information-power/data-adequacy rationale alongside saturation and discuss substantively how recruiting only engaged completers may have skewed the predominantly positive themes and limited transferability- add to limitations please. These are clarifying changes only. | The following was added to the Methods section (page 9): Sample size adequacy was evaluated using a dual framework combining a model of information power and the principle of data saturation [25,26]. Regarding information power, the study had relatively narrow and specific aims (exploring intervention integration and its barriers and facilitators) and a highly specific sample (19 participants who completed a distinct cognitive training intervention and who were rich information holders). The interview dialogue quality was strong, facilitated by the semi-structured interview guide and the cross-case analysis strategy focused on patterns within this cohort. Because of these characteristics, a moderate sample of 19 was considered sufficient. Additionally, data saturation was evaluated continuously. By the final three interviews no new codes, thematic properties, or novel barriers/facilitators emerged, confirming that informational redundancy had been reached.  The following was added to the Limitations section (pages 19-20): Because the 19 participants successfully finished the intervention, their perspectives are inherently shaped by a baseline level of motivation, adherence, and perceived value. Consequently, the data yielded predominantly positive themes while barriers experienced by non-completers remain uncaptured. This survivorship bias limits the transferability of the findings. |
