# Supplementary material for: Maintaining cognitive gains in type 2 diabetes: Barriers, supports, and patterns of strategy use
Source: PLoS One. 2026 Oct 5;21(10):e0349848. doi: 10.1371/journal.pone.0349848 (PMC13637923; doi:10.1371/journal.pone.0349848)
Supplement: Peer review history

# Peer review: Decision Letter 0

**DOI:** 10.1371/journal.pone.0349848.r001

**Authors:** Helen Howard

1 Jun 2026

PONE-D-26-06041

Maintaining cognitive gains in type 2 diabetes: Barriers, supports, and patterns of strategy use

PLOS One

Dear Dr. Cuevas,

Thank you for submitting your manuscript to PLOS ONE. After careful consideration, we feel that it has merit but does not fully meet PLOS ONE’s publication criteria as it currently stands. Therefore, we invite you to submit a revised version of the manuscript that addresses the points raised during the review process.

The manuscript has been evaluated by two reviewers, and their comments are available below.

The reviewers have raised a number of concerns that need attention. In particular, they request additional information on methodological aspects of the study, attention to the interpretation of the results, and improvements to the Discussion section.

Could you please revise the manuscript to carefully address the concerns raised?

Please submit your revised manuscript by Jul 16 2026 11:59PM. If you will need more time than this to complete your revisions, please reply to this message or contact the journal office at plosone@plos.org. When you're ready to submit your revision, log on to https://www.editorialmanager.com/pone/ and select the 'Submissions Needing Revision' folder to locate your manuscript file.

Please include the following items when submitting your revised manuscript:

- A letter that responds to each point raised by the academic editor and reviewer(s). You should upload this letter as a separate file labeled 'Response to Reviewers'.
- A marked-up copy of your manuscript that highlights changes made to the original version. You should upload this as a separate file labeled 'Revised Manuscript with Track Changes' A
- n unmarked version of your revised paper without tracked changes. You should upload this as a separate file labeled 'Manuscript'.

If you would like to make changes to your financial disclosure, please include your updated statement in your cover letter. Guidelines for resubmitting your figure files are available below the reviewer comments at the end of this letter.

If applicable, we recommend that you deposit your laboratory protocols in protocols.io to enhance the reproducibility of your results. Protocols.io assigns your protocol its own identifier (DOI) so that it can be cited independently in the future. For instructions see: https://journals.plos.org/plosone/s/submission-guidelines#loc-laboratory-protocols. Additionally, PLOS ONE offers an option for publishing peer-reviewed Lab Protocol articles, which describe protocols hosted on protocols.io. Read more information on sharing protocols at https://plos.org/protocols?utm_medium=editorial-email&utm_source=authorletters&utm_campaign=protocols.

As the corresponding author, your ORCID iD is verified in the submission system and will appear in the published article. PLOS supports the use of ORCID, and we encourage all coauthors to register for an ORCID iD and use it as well. Please encourage your coauthors to verify their ORCID iD within the submission system before final acceptance, as unverified ORCID iDs will not appear in the published article. *Only*  the individual author can complete the verification step; PLOS staff *cannot*  verify ORCID iDs on behalf of authors.

We look forward to receiving your revised manuscript.

Kind regards,

Helen Howard

Staff Editor

PLOS One

**Journal Requirements:**

When submitting your revision, we need you to address these additional requirements.

1. Please ensure that your manuscript meets PLOS ONE's style requirements, including those for file naming. The PLOS ONE style templates can be found at

https://journals.plos.org/plosone/s/file?id=wjVg/PLOSOne_formatting_sample_main_body.pdf and

https://journals.plos.org/plosone/s/file?id=ba62/PLOSOne_formatting_sample_title_authors_affiliations.pdf

2. Peer review at PLOS  One is not double-blinded (https://journals.plos.org/plosone/s/editorial-and-peer-review-process). For this reason, authors should include in the revised manuscript all the information removed for blind review.

3. Please include your full ethics statement in the ‘Methods’ section of your manuscript file. In your statement, please include the full name of the IRB or ethics committee who approved or waived your study, as well as whether or not you obtained informed written or verbal consent. If consent was waived for your study, please include this information in your statement as well.

4. If the reviewer comments include a recommendation to cite specific previously published works, please review and evaluate these publications to determine whether they are relevant and should be cited. There is no requirement to cite these works unless the editor has indicated otherwise.

[Note: HTML markup is below. Please do not edit.]

Reviewers' comments:

Reviewer's Responses to Questions

**Comments to the Author**

1. Is the manuscript technically sound, and do the data support the conclusions?

The manuscript must describe a technically sound piece of scientific research with data that supports the conclusions. Experiments must have been conducted rigorously, with appropriate controls, replication, and sample sizes. The conclusions must be drawn appropriately based on the data presented.

Reviewer #1: Yes

Reviewer #2: Partly

**********

2. Has the statistical analysis been performed appropriately and rigorously?

Reviewer #1: N/A

Reviewer #2: N/A

**********

3. Have the authors made all data underlying the findings in their manuscript fully available?

The PLOS Data policy requires authors to make all data underlying the findings described in their manuscript fully available without restriction, with rare exception (please refer to the Data Availability Statement in the manuscript PDF file). The data should be provided as part of the manuscript or its supporting information, or deposited to a public repository. For example, in addition to summary statistics, the data points behind means, medians and variance measures should be available. If there are restrictions on publicly sharing data—e.g. participant privacy or use of data from a third party—those must be specified.

Reviewer #1: No

Reviewer #2: No

**********

4. Is the manuscript presented in an intelligible fashion and written in standard English?

PLOS ONE does not copyedit accepted manuscripts, so the language in submitted articles must be clear, correct, and unambiguous. Any typographical or grammatical errors should be corrected at revision, so please note any specific errors here.

Reviewer #1: Yes

Reviewer #2: Yes

**********

5. Review Comments to the Author

Please use the space provided to explain your answers to the questions above. You may also include additional comments for the author, including concerns about dual publication, research ethics, or publication ethics. (Please upload your review as an attachment if it exceeds 20,000 characters)

Reviewer #1: Thank you for the opportunity to review this manuscript. The study addresses an important and relevant topic: how adults with type 2 diabetes maintain cognitive strategies after participating in the MAPSS-DM intervention. The qualitative approach is appropriate, and the participant quotations help support the main themes.

The manuscript has several strengths. It focuses on the actual use of cognitive strategies in daily life and identifies practical factors influencing their maintenance, including routines, flexibility, social support, and feelings of guilt or shame. These findings could be useful for improving future interventions for cognitive rehabilitation and diabetes self-management.

I recommend a revision before publication. The authors should provide more details about recruitment and sampling. In particular, it would be helpful to know if the four participants who agreed to be interviewed but ultimately did not participate differed from the nineteen participants included. Since all interviewed participants completed the intervention, the sample might represent a more motivated or adherent group.

The analysis process should also be clarified. The manuscript mentions 88% agreement among the researchers, but it is unclear how this percentage was calculated or how disagreements were resolved. Additional details on coding would enhance methodological transparency.

The results section would also benefit from a clearer distinction between the three maintenance profiles: sustained use, partial use, and discontinuation of strategies. A brief comparative table could help illustrate how barriers and facilitators differed between these groups.

Some conclusions should be formulated with greater caution. The manuscript occasionally suggests that cognitive strategies improved diabetes self-management or glycemic stability, but the data presented are primarily qualitative and self-reported. These points should therefore be presented as participants' perceptions unless objective data are added.

The STAC-r theoretical framework is relevant but could be better integrated into the discussion. It is well presented in the introduction but is underutilized in interpreting the results.

Finally, the data availability statement should include a precise link to the repository, a DOI, or an access number. The authors should also check Table 3, particularly the ethnic categories and A1C values, and correct minor language and formatting errors such as “United States of American” and “IRB#xxxx”.

Overall, this is a useful and readable qualitative manuscript. With improved methodological clarity, more careful formulation of conclusions, and correction of presentation issues, it could make a valuable contribution to the literature on cognitive rehabilitation and self-management of diabetes.

Reviewer #2: Comments to the Author

This manuscript examines how adults with type 2 diabetes maintain cognitive strategies following participation in the MAPSS-DM cognitive rehabilitation intervention. Using 3-month follow-up interviews with 19 participants, the authors identify three patterns of maintenance, sustained use, partial use, and discontinuation, and argue that long-term strategy use is shaped by daily routines, social support, emotional responses such as guilt, and perceived value beyond glycemic control. The topic is timely and clinically relevant, particularly given increasing interest in cognitive self-management strategies in chronic disease especially in diabetes. However, I have some concerns that I would like to share in order to improve the paper:

### Background: This part of the manuscript is heavily weighted toward broad biomedical background on diabetes-related cognitive decline, Alzheimer’s disease risk, and neurobiological mechanisms. While these areas are well cited, the framing becomes diffuse and at times reads more like a general narrative review than a focused rationale for a qualitative study.

For example, extended discussion of gray matter changes, insulin resistance-related brain alterations, and multiple cognitive intervention trials across different populations dilutes the central aim of the study, which is not to establish the efficacy of cognitive interventions but to understand post-intervention maintenance behavior. As a result, the actual research gap, how and why individuals with T2DM sustain or discontinue cognitive strategies in daily life, emerges relatively late and remains under-specified.

I suggest a more focused introduction that directly builds toward strategy maintenance, habit formation, and behavioral integration in diabetes self-management would strengthen conceptual clarity and better justify the qualitative approach.

#### Material and Methods: Several concerns in this section; regarding transparency and interpretive rigor. The use of a qualitative descriptive design and thematic analysis is appropriate for the research question; however, important details regarding analytic procedures are underdeveloped.

The authors state they used a “qualitative descriptive approach” and Braun & Clarke thematic analysis, but do not explain whether the analysis was inductive or deductive, semantic or latent, or what epistemological perspective guided interpretation. The analytic approach currently feels vague and procedural.Example: “Themes were identified with the empirical codes” is too general. Please explain how themes were developed and refined.

- The use of “88% agreement” is problematic in the context of Braun & Clarke thematic analysis. Reflexive thematic analysis does not typically rely on intercoder reliability statistics. This creates methodological inconsistency and suggests confusion between coding reliability approaches and reflexive thematic analysis. Example: “The researchers had a high level (88%) of agreement…” should either be justified more carefully or removed.

- Sampling and recruitment require stronger justification. Participants were recruited from intervention completers who agreed to follow-up interviews, which likely introduced selection bias toward highly engaged participants with more positive experiences. Please discuss: why 19 participants were considered adequate? ,whether thematic saturation or information power was considered? ,how non-participation may have affected findings?.

- The interviews were conducted by the primary investigator, but there is little discussion of how this may have influenced participant responses or interpretation of findings.

Participants may have felt pressure to provide favorable responses because the interviewer was connected to the intervention study.

- The interview guide appears broad and occasionally unfocused relative to the stated aims. Several questions (e.g., wake-up time, meals, leisure activities) are only indirectly related to maintenance of cognitive strategies and diabetes self-management.

- More detail is needed regarding the actual analytic process used in this study: how codes evolved ?,how disagreements were resolved?, how themes were refined?,…

- The themes appear largely positive and intervention-supportive. It is unclear whether the analysis actively explored negative experiences, disengagement, or resistance to the intervention.

- Participant characteristics for the qualitative sample are missing. Readers need demographic and clinical information to assess transferability of findings. You cite previous work ref number 25 and you blind it for the review process, regardless the demographic information is important and need to be stated in this paper.

- The manuscript claims use of the COREQ checklist to improve rigor; however, COREQ is a reporting guideline rather than a methodological strategy.

- The methods section currently mixes quantitative concepts of rigor with qualitative thematic analysis in a way that weakens methodological clarity. Greater transparency and consistency are needed.

-

### Results: Athurs need to be cautious in interpretation of the what qualitative data can support. For instance, statements such as “consistency was a main driver for maintaining strategies” or “flexibility resulted in greater maintenance” imply causal relationships that cannot be established from retrospective interviews. The findings would be more appropriately framed as perceived associations or reported influences. Similarly, while participant counts (e.g., 10 sustained users, 6 partial users, 3 discontinued) are helpful for contextualizing variation, they are occasionally treated as meaningful distributions rather than illustrative patterns, which risks overstating inferential weight in a small purposive sample.

#### Discussion: The discussion would benefit from tighter linkage between specific empirical themes and their theoretical implications, as well as clearer acknowledgment of the study’s limited transferability due to sample characteristics (highly motivated completers, older adults, and intervention participants only).

Limitations- Please address all issues that can not be resolved in the method section here.

Please discuss here – the dual role of PI as an interviewer too, and participants as both intervention completers and interviewees.

And please address the sampling limitations.

Minor Concerns-

Line 37: “ find T2DM [4], and T2DM” fix the word and.

The paper currently too long, please try to shorten it and make it easier to read.

Overall, this is a promising study with an interesting dataset and a relevant research question, but it requires substantial refinement in framing, methodological transparency, and interpretive restraint.

**********

6. PLOS authors have the option to publish the peer review history of their article (what does this mean?). If published, this will include your full peer review and any attached files.

If you choose “no”, your identity will remain anonymous but your review may still be made public.

**Do you want your identity to be public for this peer review?**  For information about this choice, including consent withdrawal, please see our Privacy Policy.

Reviewer #1: No

Reviewer #2: No

**********

[NOTE: If reviewer comments were submitted as an attachment file, they will be attached to this email and accessible via the submission site. Please log into your account, locate the manuscript record, and check for the action link "View Attachments". If this link does not appear, there are no attachment files.]

To ensure your figures meet our technical requirements, please review our figure guidelines: https://journals.plos.org/plosone/s/figures

You may also use PLOS’s free figure tool, NAAS, to help you prepare publication quality figures: https://journals.plos.org/plosone/s/figures#loc-tools-for-figure-preparation.

NAAS will assess whether your figures meet our technical requirements by comparing each figure against our figure specifications.

# Author comment: Author response to Decision Letter 1

**DOI:** 10.1371/journal.pone.0349848.r002

6 Aug 2026

Reviewer Comments

Editor

1. Please include your full ethics statement in the ‘Methods’ section of your manuscript file. In your statement, please include the full name of the IRB or ethics committee who approved or waived your study, as well as whether you obtained informed written or verbal consent. If consent was waived for your study, please include this information in your statement as well.

* Information was added (p. 6, lines 125-129; 131- 132.

Reviewer 1

1. The authors should provide more details about recruitment and sampling. It would be helpful to know if the four participants who agreed to be interviewed but ultimately did not participate differed from the nineteen participants included. Since all interviewed participants completed the intervention, the sample might represent a more motivated or adherent group.

* We added detail to the study sample section (p. 6, lines 125-129). Additionally, we discussed the possibility of increased motivation for the current sample in the limitations section (p. 19, lines 384-385).

2. The analysis process should also be clarified. The manuscript mentions 88% agreement among the researchers, but it is unclear how this percentage was calculated or how disagreements were resolved. Additional details on coding would enhance methodological transparency.

* Clarification was added to the Data Analysis section (p. 8-9, lines 167-190). Table 2 provides detail on how codes moved from participants’ words to themes to analytic categories, to broader themes.

3. The results section would also benefit from a clearer distinction between the three maintenance profiles: sustained use, partial use, and discontinuation of strategies.

* Definitions of sustained use, partial use and discontinuation of strategies were added to the results section (p. 12, lines 208-212).

4. Some conclusions should be formulated with greater caution. The manuscript occasionally suggests that cognitive strategies improved diabetes self-management or glycemic stability, but the data presented are primarily qualitative and self-reported. These points should therefore be presented as participants' perceptions unless objective data are added.

* The manuscript was edited throughout and detail specific to this concern was added to the Discussion section (p. 18, lines 345-352).

5. The STAC-r theoretical framework is relevant but could be better integrated into the discussion. It is well presented in the introduction but is underutilized in interpreting the results.

* Detail regarding the STAC-r framework was added to the discussion section (p. 17, lines 322-328; p. 17, lines 336-338; p. 18, lines 355-357, 366-368).

6. Finally, the data availability statement should include a precise link to the repository, a DOI, or an access number.

* Data is published at “MAPSS Qual Dataset.” Texas Data Repository. https://doi.org/10.18738/T8/DPNOYJ.

7. The authors should also check Table 3, particularly the ethnic categories and A1C values, and correct minor language and formatting errors.

* Formatting was corrected and errors fixed.

Reviewer 2

1. Background: This part of the manuscript is heavily weighted toward broad biomedical background on diabetes-related cognitive decline, Alzheimer’s disease risk, and neurobiological mechanisms. While these areas are well cited, the framing becomes diffuse and at times reads more like a general narrative review than a focused rationale for a qualitative study. For example, extended discussion of gray matter changes, insulin resistance-related brain alterations, and multiple cognitive intervention trials across different populations dilutes the central aim of the study, which is not to establish the efficacy of cognitive interventions but to understand post-intervention maintenance behavior. As a result, the actual research gap, how and why individuals with T2DM sustain or discontinue cognitive strategies in daily life, emerges relatively late and remains under-specified. I suggest a more focused introduction that directly builds toward strategy maintenance, habit formation, and behavioral integration in diabetes self-management would strengthen conceptual clarity and better justify the qualitative approach.

* The Background section was edited and information regarding habit formation and behavioral integration was added (p. 4, lines 77-86).

2. Material and Methods: The authors state they used a “qualitative descriptive approach” and Braun & Clarke thematic analysis, but do not explain whether the analysis was inductive or deductive, semantic or latent, or what epistemological perspective guided interpretation. The analytic approach currently feels vague and procedural. Example: “Themes were identified with the empirical codes” is too general. Please explain how themes were developed and refined.

* Clarification and details were added to the Data Analysis section (p. 8, lines 167-190). Table 2 provides detail on how codes moved from participants’ words to themes to analytic categories, to broader themes.

3. The use of “88% agreement” is problematic in the context of Braun & Clarke thematic analysis. Reflexive thematic analysis does not typically rely on intercoder reliability statistics. This creates methodological inconsistency and suggests confusion between coding reliability approaches and reflexive thematic analysis. Example: “The researchers had a high level (88%) of agreement…” should either be justified more carefully or removed.

* Reference to 88% agreement was removed.

4. Sampling and recruitment require stronger justification. Participants were recruited from intervention completers who agreed to follow-up interviews, which likely introduced selection bias toward highly engaged participants with more positive experiences. Please discuss: why 19 participants were considered adequate? whether thematic saturation or information power was considered? How non-participation may have affected findings?

* We stopped the individual interviews and focus groups after 19 participants, as we considered that saturation was achieved. We had specified in the protocol that data collection would continue until saturation point had been reached and no new domains or themes were generated (p. 12, lines 202-204). How non-participation affected findings was added to the limitations section (p. 19, lines 384-385).

5. The interviews were conducted by the primary investigator, but there is little discussion of how this may have influenced participant responses or interpretation of findings. Participants may have felt pressure to provide favorable responses because the interviewer was connected to the intervention study.

* Detail was added: To reduce influential bias, status, function and hierarchical position were not

introduced to participants and visual indication of status was purposely removed (for example lab coat and signs of academic position). No participants were under the care of the PI, who is an APN, and it was explicitly mentioned that participation in the study was disconnected with any type of medical care. (p. 7, lines 155-158).

6. The interview guide appears broad and occasionally unfocused relative to the stated aims. Several questions (e.g., wake-up time, meals, leisure activities) are only indirectly related to maintenance of cognitive strategies and diabetes self-management.

* The interview guide was kept broad to allow for flexibility and participant-driven exploration. Rather than acting as a strict, rigid script, it served as a memory aid and thematic checklist. This openness prevented missing unanticipated, emergent themes and encouraged in-depth, authentic narratives. We have added more description of the interview guide to the manuscript (p. 7, lines 152-153).

7. More detail is needed regarding the actual analytic process used in this study: how codes evolved? how disagreements were resolved? how themes were refined?

* A step-by-step guide to the analytic process has been added in the analysis section (p. 8-9, lines 167-190). Table 2 shows how the researchers moved from quotes to descriptive themes based on code groups to analytic categories to finally broad groups of analytic categories.

8. The themes appear largely positive and intervention supportive. It is unclear whether the analysis actively explored negative experiences, disengagement, or resistance to the intervention.

* We have included quotes throughout the results section (pp. 12, 14, 15) from participants who did not continue to use certain behavioral strategies. We have also added some discussion as to how this means the intervention was “less successful” or needs to be adapted/refined (p. 16).

9. Participant characteristics for the qualitative sample are missing. Readers need demographic and clinical information to assess transferability of findings. You cite previous work ref number 25 and you blind it for the review process, regardless the demographic information is important and need to be stated in this paper.

* Participant characteristics are outlined in Table 3.

10. The manuscript claims use of the COREQ checklist to improve rigor; however, COREQ is a reporting guideline rather than a methodological strategy.

* We employed multiple strategies to ensure the credibility, transferability, dependability, and confirmability of this research, and applied active reflexivity, understood as aligning three levels of reflection: researchers’ positionality, participants’ perceptions of that positionality, and the assumptions we make about those perceptions and other factors built into the qualitative interviewing and coding process. Reflexive awareness of the team’s positionality, together with investigator triangulation during coding, was used to support transparency and rigor (p. 9, lines 186-190).

11. The methods section currently mixes quantitative concepts of rigor with qualitative thematic analysis in a way that weakens methodological clarity. Greater transparency and consistency are needed.

* We have edited the methods section and added detail to the methods section (also see the above response).

Results:

12. Authors need to be cautious in interpretation of what qualitative data can support. For instance, statements such as “consistency was a main driver for maintaining strategies” or “flexibility resulted in greater maintenance” imply causal relationships that cannot be established from retrospective interviews. The findings would be more appropriately framed as perceived associations or reported influences. Similarly, while participant counts (e.g., 10 sustained users, 6 partial users, 3 discontinued) are helpful for contextualizing variation, they are occasionally treated as meaningful distributions rather than illustrative patterns, which risks overstating inferential weight in a small purposive sample.

* We have tempered the causal language throughout the results section where appropriate (p. 13, lines 223-224, 236; p. 14, lines 244, 250, 255; p. 15, lines 274. 282, 286).

13. Discussion: The discussion would benefit from tighter linkage between specific empirical themes and their theoretical implications, as well as clearer acknowledgment of the study’s limited transferability due to sample characteristics (highly motivated completers, older adults, and intervention participants only).

* We have revised the discussion section and improved these linkages as well as expanded the limitations section (p. 17, lines 322-328; p. 17, lines 336-338; p. 18, lines 355-357, 366-368).

Limitations

14. Please address all issues that cannot be resolved in the method section here. Please discuss here – the dual role of PI as an interviewer too, and participants as both intervention completers and interviewees. And please address the sampling limitations.

* We have expanded the limitations section as well as included more detail in the methods section discussing these issues (p. 6, lines 125-129; p. 7, lines 155-158; p. 19, lines 382-387).

15. Minor Concerns-Line 37: “find T2DM [4], and T2DM” fix the word and.

* Corrected.

16. he paper currently too long, please try to shorten it and make it easier to read.

* We have edited and shortened the paper where appropriate.

Attachment Submitted filename: MAPSS Qual Response to Reviewers.docx

# Peer review: Decision Letter 1

**DOI:** 10.1371/journal.pone.0349848.r003

**Authors:** Ayman Swelum

30 Aug 2026

Dear Dr. Cuevas,

Thank you for submitting your manuscript to PLOS One. After careful consideration, we feel that it has merit but does not fully meet PLOS One’s publication criteria as it currently stands. Therefore, we invite you to submit a revised version of the manuscript that addresses the points raised during the review process.

Please submit your revised manuscript by Oct 14 2026 11:59PM. If you will need more time than this to complete your revisions, please reply to this message or contact the journal office at plosone@plos.org. When you're ready to submit your revision, log on to https://www.editorialmanager.com/pone/ and select the 'Submissions Needing Revision' folder to locate your manuscript file.

- A letter that responds to each point raised by the academic editor and reviewer(s). You should upload this letter as a separate file labeled 'Response to Reviewers'.
- A marked-up copy of your manuscript that highlights changes made to the original version. You should upload this as a separate file labeled 'Revised Manuscript with Track Changes'.
- An unmarked version of your revised paper without tracked changes. You should upload this as a separate file labeled 'Manuscript'.

If you would like to make changes to your financial disclosure, please include your updated statement in your cover letter. Guidelines for resubmitting your figure files are available below the reviewer comments at the end of this letter.

If applicable, we recommend that you deposit your laboratory protocols in protocols.io to enhance the reproducibility of your results. Protocols.io assigns your protocol its own identifier (DOI) so that it can be cited independently in the future. For instructions see: https://journals.plos.org/plosone/s/submission-guidelines#loc-laboratory-protocols. Additionally, PLOS One offers an option for publishing peer-reviewed Lab Protocol articles, which describe protocols hosted on protocols.io. Read more information on sharing protocols at https://plos.org/protocols?utm_medium=editorial-email&utm_source=authorletters&utm_campaign=protocols.

As the corresponding author, your ORCID iD is verified in the submission system and will appear in the published article. PLOS supports the use of ORCID, and we encourage all coauthors to register for an ORCID iD and use it as well. Please encourage your coauthors to verify their ORCID iD within the submission system before final acceptance, as unverified ORCID iDs will not appear in the published article. *Only*  the individual author can complete the verification step; PLOS staff *cannot*  verify ORCID iDs on behalf of authors.

We look forward to receiving your revised manuscript.

Kind regards,

Ayman A Swelum

Academic Editor

PLOS One

Journal Requirements:

If the reviewer comments include a recommendation to cite specific previously published works, please review and evaluate these publications to determine whether they are relevant and should be cited. There is no requirement to cite these works unless the editor has indicated otherwise.

Please review your reference list to ensure that it is complete and correct. If you have cited papers that have been retracted, please include the rationale for doing so in the manuscript text, or remove these references and replace them with relevant current references. Any changes to the reference list should be mentioned in the rebuttal letter that accompanies your revised manuscript. If you need to cite a retracted article, indicate the article’s retracted status in the References list and also include a citation and full reference for the retraction notice.

[Note: HTML markup is below. Please do not edit.]

Reviewers' comments:

Reviewer's Responses to Questions

**Comments to the Author**

Reviewer #1: All comments have been addressed

Reviewer #2: All comments have been addressed

**********

2. Is the manuscript technically sound, and do the data support the conclusions?

Reviewer #1: Yes

Reviewer #2: Yes

**********

3. Has the statistical analysis been performed appropriately and rigorously?

Reviewer #1: N/A

Reviewer #2: I Don't Know

**********

4. Have the authors made all data underlying the findings in their manuscript fully available?

The PLOS Data policy

Reviewer #1: Yes

Reviewer #2: Yes

**********

5. Is the manuscript presented in an intelligible fashion and written in standard English?

Reviewer #1: Yes

Reviewer #2: Yes

**********

Reviewer #1: Thank you for carefully considering the comments made in my previous review. The revisions have improved the methodological transparency and clarity of the manuscript, particularly with regard to recruitment and sampling, the qualitative analysis process, the distinction between maintenance profiles, the interpretation of the findings, the integration of the STAC-r framework, and data availability. My previous concerns have been satisfactorily addressed, and I have no further significant comments.

Reviewer #2: Dear Authors,

Thank you for your thorough revisions, which have substantially improved the manuscript.

Before I can recommend acceptance, please confirm or make explicit one minor items:

Add an information-power/data-adequacy rationale alongside saturation, and discuss substantively how recruiting only engaged completers may have skewed the predominantly positive themes and limited transferability- add to limitations please.

These are clarifying changes only. Once confirmed, I would be pleased to support acceptance.

Sincerely, Reviewer 2

**********

what does this mean?). If published, this will include your full peer review and any attached files.

If you choose “no”, your identity will remain anonymous but your review may still be made public.

**Do you want your identity to be public for this peer review?**  For information about this choice, including consent withdrawal, please see our Privacy Policy

Reviewer #1: No

Reviewer #2: No

**********

[NOTE: If reviewer comments were submitted as an attachment file, they will be attached to this email and accessible via the submission site. Please log into your account, locate the manuscript record, and check for the action link "View Attachments". If this link does not appear, there are no attachment files.]

To ensure your figures meet our technical requirements, please review our figure guidelines: https://journals.plos.org/plosone/s/figures

You may also use PLOS’s free figure tool, NAAS, to help you prepare publication quality figures: https://journals.plos.org/plosone/s/figures#loc-tools-for-figure-preparation.

NAAS will assess whether your figures meet our technical requirements by comparing each figure against our figure specifications.

# Author comment: Author response to Decision Letter 2

**DOI:** 10.1371/journal.pone.0349848.r004

2 Sep 2026

Reviewer Comments

Add an information-power/data-adequacy rationale alongside saturation and discuss substantively how recruiting only engaged completers may have skewed the predominantly positive themes and limited transferability- add to limitations please.

These are clarifying changes only.

Response

The following was added to the Methods section (page 9): Sample size adequacy was evaluated using a dual framework combining a model of information power and the principle of data saturation [25,26]. Regarding information power, the study had relatively narrow and specific aims (exploring intervention integration and its barriers and facilitators) and a highly specific sample (19 participants who completed a distinct cognitive training intervention and who were rich information holders). The interview dialogue quality was strong, facilitated by the semi-structured interview guide and the cross-case analysis strategy focused on patterns within this cohort. Because of these characteristics, a moderate sample of 19 was considered sufficient. Additionally, data saturation was evaluated continuously. By the final three interviews no new codes, thematic properties, or novel barriers/facilitators emerged, confirming that informational redundancy had been reached.

The following was added to the Limitations section (pages 19-20): Because the 19 participants successfully finished the intervention, their perspectives are inherently shaped by a baseline level of motivation, adherence, and perceived value. Consequently, the data yielded predominantly positive themes while barriers experienced by non-completers remain uncaptured. This survivorship bias limits the transferability of the findings.

Attachment Submitted filename: Response to Reviewers 2.docx

# Peer review: Decision Letter 2

**DOI:** 10.1371/journal.pone.0349848.r005

**Authors:** Ayman Swelum

18 Sep 2026

Maintaining cognitive gains in type 2 diabetes: Barriers, supports, and patterns of strategy use

PONE-D-26-06041R2

Dear Dr. Cuevas,

We’re pleased to inform you that your manuscript has been judged scientifically suitable for publication and will be formally accepted for publication once it meets all outstanding technical requirements.

Within one week, you’ll receive an e-mail detailing the required amendments. When these have been addressed, you’ll receive a formal acceptance letter and your manuscript will be scheduled for publication.

An invoice will be generated when your article is formally accepted. Please note, if your institution has a publishing partnership with PLOS and your article meets the relevant criteria, all or part of your publication costs will be covered. Please make sure your user information is up-to-date by logging into Editorial Manager at Editorial Manager® and clicking the ‘Update My Information' link at the top of the page. For questions related to billing, please contact billing support.

If your institution or institutions have a press office, please notify them about your upcoming paper to help maximize its impact. If they’ll be preparing press materials, please inform our press team as soon as possible -- no later than 48 hours after receiving the formal acceptance. Your manuscript will remain under strict press embargo until 2 pm Eastern Time on the date of publication. For more information, please contact onepress@plos.org.

Kind regards,

Ayman A Swelum

Academic Editor

PLOS One

Additional Editor Comments (optional):

Reviewers' comments:

Reviewer's Responses to Questions

**Comments to the Author**

Reviewer #2: All comments have been addressed

**********

2. Is the manuscript technically sound, and do the data support the conclusions?

Reviewer #2: Yes

**********

3. Has the statistical analysis been performed appropriately and rigorously?

Reviewer #2: I Don't Know

**********

4. Have the authors made all data underlying the findings in their manuscript fully available?

The PLOS Data policy

Reviewer #2: No

**********

5. Is the manuscript presented in an intelligible fashion and written in standard English?

Reviewer #2: Yes

**********

Reviewer #2: Congratulations to all coauthors

Heather Cuevas

Jeeyeon Kim, PhD

Shenell T Wood, PhD

I have no further comments.

**********

what does this mean?). If published, this will include your full peer review and any attached files.

If you choose “no”, your identity will remain anonymous but your review may still be made public.

**Do you want your identity to be public for this peer review?**  For information about this choice, including consent withdrawal, please see our Privacy Policy

Reviewer #2: No

**********

# Editor report: Acceptance letter

**DOI:** 10.1371/journal.pone.0349848.r006

**Authors:** Ayman Swelum

PONE-D-26-06041R2

PLOS One

Dear Dr. Cuevas,

I'm pleased to inform you that your manuscript has been deemed suitable for publication in PLOS One. Congratulations! Your manuscript is now being handed over to our production team.

At this stage, our production department will prepare your paper for publication. This includes ensuring the following:

* All references, tables, and figures are properly cited

* All relevant supporting information is included in the manuscript submission,

* There are no issues that prevent the paper from being properly typeset

You will receive further instructions from the production team, including instructions on how to review your proof when it is ready. Please keep in mind that we are working through a large volume of accepted articles, so please give us a few days to review your paper and let you know the next and final steps.

Lastly, if your institution or institutions have a press office, please let them know about your upcoming paper now to help maximize its impact. If they'll be preparing press materials, please inform our press team within the next 48 hours. Your manuscript will remain under strict press embargo until 2 pm Eastern Time on the date of publication. For more information, please contact onepress@plos.org.

You will receive an invoice from PLOS for your publication fee after your manuscript has reached the completed accept phase. If you receive an email requesting payment before acceptance or for any other service, this may be a phishing scheme. Learn how to identify phishing emails and protect your accounts at https://explore.plos.org/phishing.

If we can help with anything else, please email us at customercare@plos.org.

Thank you for submitting your work to PLOS One and supporting open access.

Kind regards,

PLOS One Editorial Office Staff

on behalf of

Professor Ayman A Swelum

Academic Editor

PLOS One
